# Genome-Wide Comparative Analysis of *Aspergillus fumigatus* Strains: The Reference Genome as a Matter of Concern

**DOI:** 10.3390/genes9070363

**Published:** 2018-07-19

**Authors:** Rocio Garcia-Rubio, Sara Monzon, Laura Alcazar-Fuoli, Isabel Cuesta, Emilia Mellado

**Affiliations:** 1 Mycology Reference Laboratory, National Centre for Microbiology, Instituto de Salud Carlos III (ISCIII), Majadahonda, 28220 Madrid, Spain; rgarciar@isciii.es (R.G.-R.); lalcazar@isciii.es (L.A.-F.); 2 Bioinformatics Unit, Common Scientific Technical Units, ISCIII, Majadahonda, 28220 Madrid, Spain; smonzon@isciii.es (S.M.); isabel.cuesta@isciii.es (I.C.)

**Keywords:** *Aspergillus fumigatus*, whole genome sequencing, reference genomes, azole resistance, *cyp51A*

## Abstract

*Aspergillus fumigatus* is a ubiquitous saprophytic mold and a major pathogen in immunocompromised patients. The effectiveness of triazole compounds, the *A. fumigatus* first line treatment, is being threatened by a rapid and global emergence of azole resistance. Whole genome sequencing (WGS) has emerged as an invaluable tool for the analysis of genetic differences between *A. fumigatus* strains, their genetic background, and antifungal resistance development. Although WGS analyses can provide a valuable amount of novel information, there are some limitations that should be considered. These analyses, based on genome-wide comparative data and single nucleotide variant (SNV) calling, are dependent on the quality of sequencing, assembling, the variant calling criteria, as well as on the suitable selection of the reference genome, which must be genetically close to the genomes included in the analysis. In this study, 28 *A. fumigatus* genomes sequenced in-house and 73 available in public data bases have been analyzed. All genomes were distributed in four clusters and showed a variable number of SNVs depending on the genome used as reference (Af293 or A1163). Each reference genome belonged to a different cluster. The results highlighted the importance of choosing the most suitable *A. fumigatus* reference genome to avoid misleading conclusions.

## 1. Introduction

*Aspergillus fumigatus* is a saprophytic filamentous fungus and the principal causative agent of human aspergillosis [1,2]. There are many types of diseases caused by *A. fumigatus* and their symptoms vary according to the site of infection and host health condition. Predominantly, this species causes invasive infections, such as invasive pulmonary aspergillosis (IPA), with high mortality rates in immunocompromised or immunosuppressed patients [3,4,5]. Currently, the treatment options are limited to three classes of antifungal drugs (azoles, echinocandins and polyenes) and azoles are drugs of first line for treating *Aspergillus*-caused diseases. However, azole drug efficacy is threatened by the worldwide spreading of resistance [6].

Despite all the technological improvements developed in recent years, many basic aspects about the biology of this opportunistic pathogen remained largely unknown. Genomics and whole genome sequencing (WGS) have emerged as useful tools to greatly enhance knowledge and understanding of infectious diseases and clinical microbiology [7,8,9,10]. In this context, genome-wide sequencing using high throughput sequencing (HTS) together with alignment comparison analysis has been described as a good approach to identify single nucleotide variants (SNVs) or polymorphisms (SNPs) for the analysis of genetic differences between *A. fumigatus* strains [11]. The genetic diversity between isolates can be used to explain many different aspects such as the strain background lineage, strain specific virulence phenotypes, the potential factors involved in antifungal resistant development, and more commonly, determining the genetic strain relatedness in epidemiological surveillance and outbreak studies [12,13,14].

Although WGS studies provide a valuable amount of novel data, there are some considerations that should be taken into account. To date, most WGS analyses are based on identifying SNVs in a set of strains in comparison to a reference genome, and consequently the results will be completely dependent on the chosen reference. Therefore, the identified SNVs will have a different significance depending on how genetically close the reference genome will be from the strains included in the analysis. Classically, most advances have been focused on unravelling the genomics of two different *A. fumigatus* reference strains [15], Af293 (AAHF00000000.1) and A1163 (ABDB00000000.1), or their derivatives. First, the Af293 strain was fully sequenced in 2005 [16], and soon after, the A1163 strain was the second *A. fumigatus* genome sequenced by the J. Craig Venter Institute [17]. This strain was a derivative of another clinical isolate, the CBS144-89/CEA10. The first genome comparison between both strains showed that despite high synteny and identity in most of the regions of their genomes, there were some hundreds of genes unique from each strain and not present in the other [18]. This heterogeneity could potentially lead to confusion, so care must be taken in drawing conclusions on different aspects from a single genetic background [15]. A recent study carried out in our laboratory [19] showed that the Af293 reference genome belonged to a specific lineage of *A. fumigatus* strains which harbors five polymorphisms (F46Y, M172V, N248T, D255E, E427K) in *cyp51A* gene which codes for the azole target. Based on these phylogenetic studies from whole genome sequencing, the other classic reference genome, the A1163 strain, belonged to a different and distant phylogenetic lineage, which implies that both genomes are significantly different. Also, and apart from the relevance of choosing the most suitable reference genome, the quality of sequencing and assembling will be of great importance for getting reliable results.

In this work, we describe the in-house genome sequencing and comparative analysis of 28 *A. fumigatus* genomes, including the *A. fumigatus* genomes of six strains routinely used in *Aspergillus* basic research laboratories. All these genomes were distributed in four separate clusters, corresponding to very different lineages. In addition, 73 *A. fumigatus* genomes, including azole susceptible and resistant strains from a worldwide distribution, were included in the study. The whole sequence analysis of these 101 *A. fumigatus* genomes provided information that led to an increase in the knowledge of *A. fumigatus* genetic background and the development of azole resistance.

## 2. Materials and Methods

### 2.1. Aspergillus fumigatus Isolates

A total of 169 genomes were evaluated including in-house sequenced *Aspergillus fumigatus* isolates and genomes downloaded from NCBI Sequence Read Archive (SRA) public database (www.ncbi.nlm.nih.gov/sra). The genomic samples were selected following this criteria: (i) all genomes had to be sequenced with Illumina platform; and (ii) had to be paired-end. After quality control filtering (>85% mapping rate against Af293 reference genome and >95% of genome coverage at more than 10× depth of coverage), a total of 101 *A. fumigatus* strain genome sequences representing 8 different geographical locations were selected for further analysis. Among them, 28 *A. fumigatus* strains were from the Mycology Reference Laboratory Collection (Table 1). Most of them were clinical azole susceptible strains. Some of them had specific *cyp51A* modifications: 12 strains harbored three changes (F46Y, M172V, and E427K) and 3 strains had five (F46Y, M172V, N248T, D255E and E427K) [19]. Af293 was included in the latter group of five *cyp51A* modifications and sequenced again in-house (named as AF293). Another group of *A. fumigatus* azole susceptible strains were isolates that have been classically used as reference or control strains in most *Aspergillus* laboratories. This set of strains included the A1163 (also known as CEA10, CBS144.89, FGSC A1163 or AF10), AF237 (called CM237), ATCC204305, *akuB*^KU80^ (FGSC A1160) [20], and ATCC46645. Two azole resistant strains without modifications in *cyp51A* were also included and sequenced in this work (CM7510 and CM7555).

The remaining 73 strain genomes were obtained from public data bases and belonged to strains from the United Kingdom, the Netherlands, Portugal, Denmark, India, Japan and Canada [13,21,22] (NCBI SRA public database) (Table 2). Some of the isolates from The Netherlands and India came from clinical samples (*n* = 8) and others from environmental soil sources (*n* = 8). Strains from Japan, UK and Canada were all from clinical origin while the Portuguese strains had an unknown origin (Table 2).

### 2.2. DNA Extraction and Aspergillus fumigatus Identification

First, conidia from each selected strain were cultured in 3 mL of glucose-yeast extract-peptone (GYEP) broth (2% glucose, 0.3% yeast extract, 1% peptone; OXOID LTD, Basingstoke, Hampshire, England) and grown overnight at 37 °C, after which mycelium mats were harvested and DNA was extracted [23]. The *A. fumigatus* Spanish isolates included in this work were identified to the species level on the basis of PCR amplification and sequencing of the internal transcribed spacer (ITS) region and the partial amplification of the *β-tubulin* gene [24].

### 2.3. DNA Quality and Quantity Assessment

The DNA quality and quantity was assessed using a spectrophotometer (Thermo Scientific NanoDrop One Spectrophotometer, Waltham, MA, USA). Since genomic DNA (gDNA) must be clean and pure for preparing WGS libraries, when the 260/280 or 260/230 rates were lower than 1.8 or higher than 2.2, the DNA was rejected. The strain was extracted again from a new mycelium mat and its quality was assessed. Genomic DNA (gDNAs) were stored at −20 °C until further use.

### 2.4. Screening of cyp51A Changes: PCR Amplification and Sequence Analysis

The full coding sequences of *cyp51A* including its promoter, were amplified and sequenced using the PCR conditions described before [25]. To exclude the possibility that any change identified in the sequences was due to PCR-induced errors, each isolate was independently analyzed twice. Isolates were screened for the presence of tandem repeat insertions in the *cyp51A* promoter region, as well as for the presence of other *cyp51A* modifications. All the nucleotide sequences were analyzed using the DNASTAR Lasergene package (DNASTAR Inc., Madison, WI, USA).

### 2.5. DNA Library Preparation and Illumina Whole-Genome Sequencing

Genomic DNA was extracted as previously described. Genomic DNA were quantified afterwards using the QuantiFluor ^®^ dsDNA System and the QuantiFluor ^®^ ST Fluorometer (Promega, Madison, WI, USA) and their quality was determined with the Agilent 2100 Bioanalyzer (Agilent Technologies, Inc., Santa Clara, CA, USA). The preparation of fragmented gDNA libraries was performed using Nextera ^®^ XT Library Prep Kit (Illumina Inc., San Diego, CA, USA), according to the manufacturer’s protocols. The mean fragment length of the libraries ranged from 800 to 1800 bp. Sequencing was conducted in paired-end 2 × 150 bp on a NextSeq 500 system, according to the manufacturer’s protocols (Illumina Inc., San Diego, CA, USA).

### 2.6. Whole-Genome Sequencing Alignment

The Illumina reads were trimmed using Trimmomatic (version 0.32) [26]. The sequencing adapters and sequences with low quality scores on 3′ ends (Phred score [Q], <20) were trimmed. Raw Illumina WGS reads were quality checked performing a quality control with FastQC (version 0.11.3; Babraham Institute). Data sets were analyzed against two different *A. fumigatus* reference genomes, the Af293 (GenBank accession number AAHF00000000.1) and the A1163 (GenBank accession number ABDB00000000.1) using WGS-outbreaker v1.0 (Instituto de Salud Carlos III, Madrid, Spain) (https://github.com/BU-ISCIII/WGS-Outbreaker) with default parameters. The pipeline comprised all steps needed for SNV analysis using whole genome sequencing data. Mapping against genome reference was performed with bwa mem (version 0.7.12-r1039) [27], duplicated reads removed using Picard (version 1.140) (http://broadinstitute.github.io/picard), and the bedtools coverage v2.26 program [28] was used to perform further quality controls. Hereafter, in order to identify genetic variations among strains, single nucleotide variant (SNV) detection (variant calling) and SNV matrix generation were performed using GATK version 3.8.0 [29] with best practices parameters. ENSEMBL variant effect predictor script (version 88) was used for variant annotation. The whole genome sequencing project has been deposited in NCBI SRA (project accession number SRP151231).

### 2.7. Phylogenetic Analysis and Single Nucleotide Variant Comparison

Final step of WGS-Outbreaker pipeline comprised Maximum-likelihood trees construction using RaxML software (version 8.2.9) [30] with GTRCAT model and 100 bootstrap replicates. Phylogenetic trees were visualized and annotated using ggtree R package [31]. SNV comparisons were performed using a custom R script, mapping all genomes to Af293 and A1163 reference genomes.

### 2.8. Analysis of Genetic Diversity

Genetic diversity was calculated using the Molecular Evolutionary Genetics Analysis software (MEGA, version 7) [32]. The *A. fumigatus* population was analyzed according to the formed groups (clusters and subclusters) from the previous phylogenic studies. In MEGA, evolutionary distances between sequences can be estimated by computing the number or proportion of nucleotide differences between sequences using the FASTA alignment against each reference genome [33]. Two different models have been used in this work, the p-distance model and another based on the number of differences; (i) the p-distance model calculates the distance as the proportion of different nucleotide sites compared. It is obtained by dividing the number of nucleotide differences (transitions and transversions) by the total number of nucleotides compared. As recommended [34], when the genetic distance was estimated, the complete-deletion option was used, since it normalizes the number of differences based on the number of valid sites compared, not taking into account the alignment gaps and missing data that sequences could contain. However, this model does not make any correction for multiple substitutions at the same site, substitution rate biases (for example, differences in the transitional and transversional rates), or differences in evolutionary rates among sites; (ii) The second model, based on the number of differences, estimates the genetic distances according to the number of sites at which the compared sequences differ (transitions and transversions). In this model, the complete-deletion option was also used.

### 2.9. Determining Modifications in Genes of Interest

In order to see if the population structure could be based on particular genomic modifications, some genes that have already been described as important in *A. fumigatus* biology were analyzed in depth in each of the *A. fumigatus* population clusters formed from the SNV comparisons. One of the genes was the *cyp51A* gene including its promoter (AFUA_4G06890 in Af293, and AFUB_063960 in A1163). This region was used to determine the azole resistant phenotype based on the resistance mechanism. Since both reference genomes, Af293 and A1163, have opposite mating types, the mating locus genes were included in the analysis: Mat1.1.1 (AFUB_042900), Mat1.2.1 (AFUA_3G06170), and Mat1.2.4 (AFUA_3G06160). The presence or absence of these loci was also tested in all genomes using srst2 software (version 0.1.8) [35]. Every gene of interest was analyzed independently in all genomes.

### 2.10. Visualization of Depth of Coverage

An exploratory approach using Circos (v 0.69.3) [36], a tool to represent visual data, was used to plot the depth of coverage of 20 whole-sequenced genomes selected to represent each cluster and subcluster in comparison to the Af293 reference genome. Mean coverage data in 10,000 length bins was calculated from bam files with bedtools (version 2.2.17) (http://bedtools.readthedocs.io/en/latest/).

### 2.11. Mating Type Analysis

A mapping approach was used in order to identify genes associated with each mating type. Since Af293 was Mat1.2 and A1163 was Mat1.1, all samples were mapped against both references using the workflow displayed in Figure S1: (i) samples that had been determined with srst2 as Mat1.2 and mapped against A1163 were selected, and unmapped reads were retained in this step; (ii) the unmapped reads were then mapped against Af293. Statistics of coverage and depth of coverage were calculated for all genes annotated in A1163 reference genome using the bam generated in this step; (iii) genes with at least 70% of coverage were selected; (iv) to discard genes that may be found due to the lack of presence in the A1163 assembly, the same procedure was performed for Mat1.1 samples; (v) finally, genes in common among all Mat1.2 samples minus one but not present in any Mat1.1 sample were exported as a result (Figure S1A). The reverse procedure was performed starting with Mat1.1 samples determining the genes only present in Mat1.1 samples (Figure S1B).

## 3. Results

### 3.1. Species Identification

The isolates were identified at the species level and also the azole resistance mechanism was analyzed by PCR amplification and sequencing of the *cyp51A* gene and its promoter as explained in Materials and Methods section. All the strains were identified as *A. fumigatus* sensu stricto. The azole susceptibility profile of most of the strains sequenced in the laboratory is described elsewhere [19]. For the remaining strains, the azole susceptibility profile was determined based on the known *cyp51A* modifications detected by WGS analysis. According to the azole resistance mechanism, the *A. fumigatu*s strains included in this study harbored the following *cyp51A* modifications: (i) strains with *cyp51A* single point mutations (G54E/R, M220V/T/K, and G138C) or double point mutations (G448S together with H147Y) in the minority of cases (only one strain); or (ii) strains with tandem integrations and *cyp51A* modifications (TR34/L98H, TR34/L98H/S297T/F495I). Two azole resistant *cyp51A* wild type (WT) strains were also included. All these strain features (*cyp51A* modifications and azole susceptibility profile, as well as the geographical origin and mating type), were described in Table 1 and Table 2. Afterwards, the DNAs were further analyzed using WGS.

### 3.2. Whole-Genome Sequencing Analysis

All genome sequences mapped to a range of 85–99% genome coverage with at least 10x of depth of coverage and a mapping rate > 94% against Af293 reference genome. Similarly, sequences were mapped to a range of 84–99% genome coverage with at least 10× of depth of coverage against A1163 reference with a mapping rate > 88% (Table S1). The Af293 and the A1163 strains were resequenced in our laboratory (and named hereafter AF293 and CEA10) and had a total of 313 and 1009 SNVs, respectively, compared to their respective reference genomes.

### 3.3. Phylogenetic Analysis

**Figure 1:**
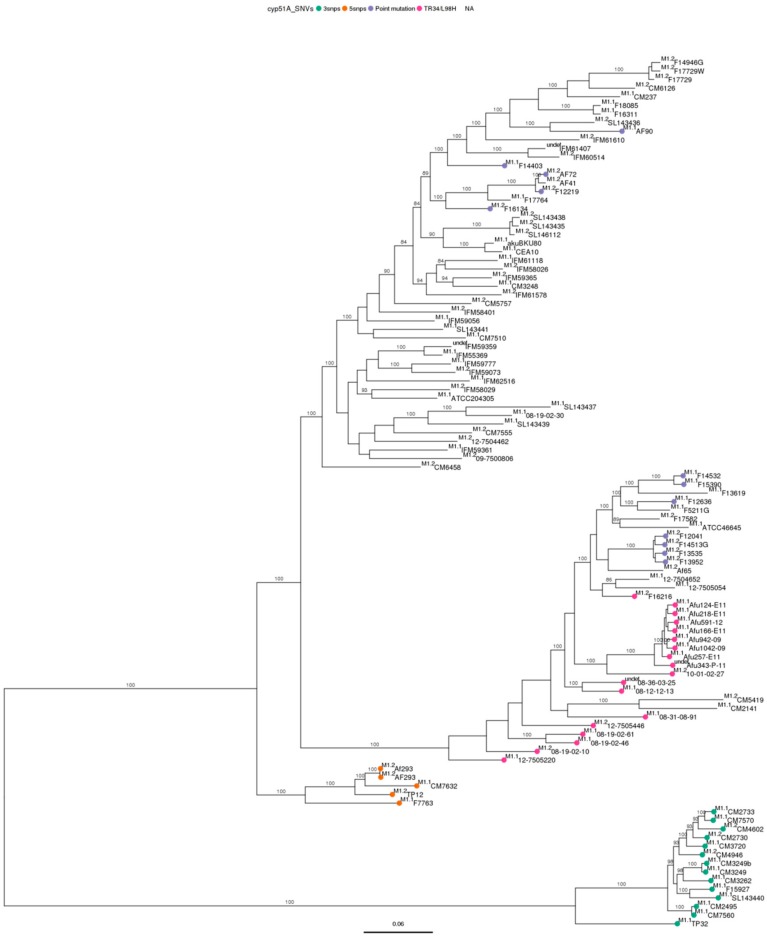
Whole genome phylogenetic analysis of 101 *Aspergillus fumigatus* genomes included in the study. Dendrogram was performed using Af293 as reference genome.

**Figure 2:**
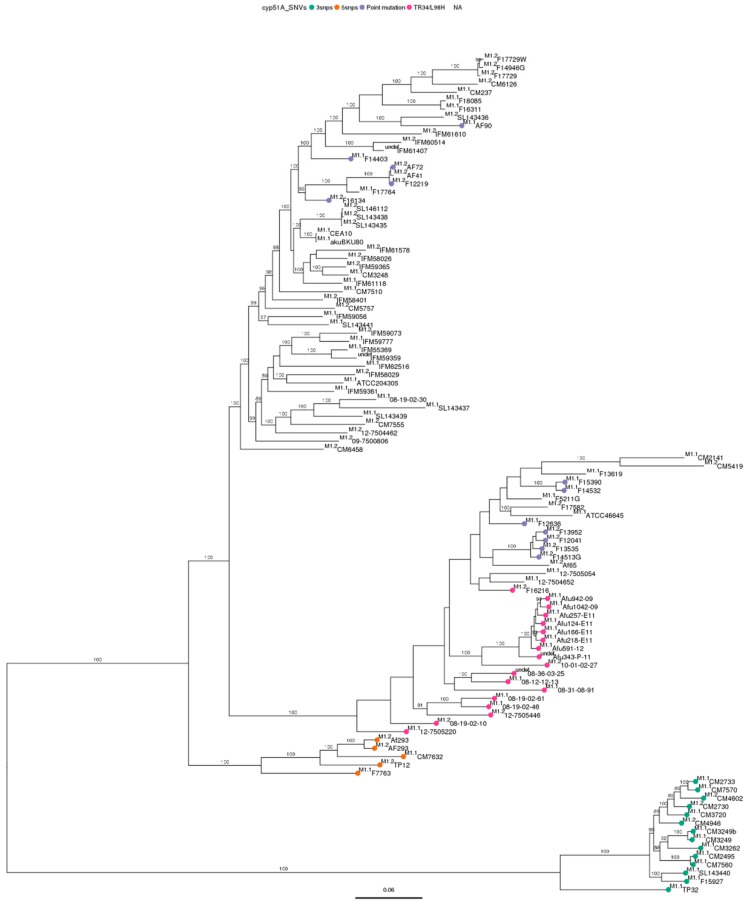
Whole genome phylogenetic analysis of 101 *Aspergillus fumigatus* genomes included in the study. Dendrogram was performed using A1163 as reference genome.

Phylogenetic analysis using SNV data of Spanish genomes (Table 1) clearly showed that our strains were divided into four clusters independently of the genome used as reference (Figure 1, with Af293 as reference genome and Figure 2, with A1163 as reference genome). In this previous analysis, the different wild type reference strains used, *akuB*^KU80^ and CEA10 as derivative strains of A1163, AF293, CM237, ATCC204305 and ATCC46645 were located in separated clusters (Figure 1 and Figure 2). Those clusters were further confirmed when the remaining 73 *A. fumigatus* genomes available from other countries were included.

Therefore, based on phylogenetic analysis, the complete *A. fumigatus* population was divided into four well defined clusters (I, II, III, and IV). Two of them could be further subdivided into subclusters: cluster I split in 3 subclusters (I.1, I.2, I.3), and cluster II in 2 (II.1, II.2). Clusters III and IV remained undivided, because any subcluster was identified. Each subcluster was considered as an independent population group, and was used to compare itself against each reference genome (Table 3). The two reference genomes used were distributed in different clusters when compared against the other: A1163 in cluster I and Af293 in cluster III. The number of clusters and the tree topology was maintained regardless of the reference genome chosen.

Based on *cyp51A* modifications, azole susceptible *cyp51A*-WT strains together with azole resistant *cyp51A* single point mutation strains were grouped together in cluster I. In cluster II, there were azole susceptible and resistant strains, with both *cyp51A* single point mutations and TR_34_/L98H mechanisms. In cluster III, a set of strains with five *cyp51A* modifications (F46Y, M172V, N248T, D255E, E427K) grouped together. While in cluster IV strains with three *cyp51A* modifications (F46Y, M172V, E427K) also grouped together [19]. The Netherlands, Portugal and Canada genomes were distributed in two clusters. The United Kingdom genomes were distributed in three, while Japanese genomes (*cyp51A*-WT) were only in cluster I and Indian genomes (*cyp51A*-TR_34_) in cluster II. Except for the tightly clustered isolates from India in cluster II.2, there was no relationship between the geographical origin of the isolates and the cluster where they grouped.

### 3.4. Single Nucleotide Variant Analysis against Both References

To explore the genomic differences between the clusters formed from phylogenetic studies, total SNVs between populations were determined. There were a total of 93,609 and 71,844 SNV positions identified against the reference genomes, Af293 and A1163, respectively. No noticeable differences were found in the SNV ranges when all strains were mapped against Af293 (313–165,788) or A1163 (1043–163,367). However, the number of specific SNVs identified in each strain was notably different depending on the genome that was used as reference (Table 4). In fact, some particular SNVs previously detected in our laboratory by PCR (L98H, F46Y, M172V, N248T, D255E, E427K) were specifically searched for in both reference whole genomes. The L98H variant was found in all genomes that harbored this mutation since none of the reference genomes had this change in its own genome. However, the remaining 5 SNVs (F46Y, M172V, N248T, D255E, E427K) were only detected when the reference genome chosen was the A1163 genome, as Af293 specifically harbored these 5 same polymorphisms.

Although the tree topology was maintained regardless of the reference genome chosen, the number of SNVs within clusters changed significantly when comparing each reference against each cluster, showing that both reference genomes were genetically very different (Table 3). The number of SNVs of clusters II and IV were similar against both references, while remarkable differences in SNVs were present in clusters I (closer to A1163) and III (closer to Af293) (Table 3). Considering subclusters, the number of SNVs in subclusters I.1, I.2 and I.3 were almost half when the reference genome was the A1163, than the SNVs obtained using Af293 as reference genome. Another remarkable result was the small number of SNVs found in cluster III when compared to Af293 genome. The SNV differences found against both references were slighter but also considerable in cluster II, higher when compared to A1163. Cluster IV had the greatest number of SNVs, independent of the reference genome used.

Moreover, in order to explore any genomic differences between clusters and subclusters, some features of the SNVs found in each population were determined (Table 5). This table shows that all populations had a similar amount of SNVs, excluding cluster IV, which had much more variants, specifically missense and synonymous, compared to the other clusters.

### 3.5. Genetic Diversity

Evolutionary distances were estimated by computing the mean proportion or number of nucleotide differences between each subcluster and each reference genome using two different models provided by MEGA: the p-model and the number of differences model.

The pairwise distance matrices referred to the distance between each genome and the others in pairs and was calculated and expressed in proportion and number of differences (Tables S2–S5). The matrices obtained from the mean distance between groups in which each subcluster was compared to each other, were indicated also as the proportion and number of differences of all these comparisons (Tables S6 and S7). Cluster IV was identified as the most distant cluster, according to phylogenetic results, with 123,259, 118,271 and 114,815 differences in SNVs against cluster I, II and III, respectively. Moreover, while cluster III was more similar to cluster II with 36,031 differences, cluster I was closer to cluster II than to III with 38,197 and 50,040 differences, respectively. These data were calculated using Af293 genome as reference. Data using A1163 were not shown since no significant differences were found.

Homogeneity within a group or cluster could be inferred through the analysis of the mean distance within groups (Tables S8 and S9). Cluster IV was the most homogeneous since the number of differences was lower than in the remaining groups, followed by subcluster II.1, independently of the genome that was chosen as reference.

### 3.6. Genome-Wide Visualization of Depth of Coverage

**Figure 3:**
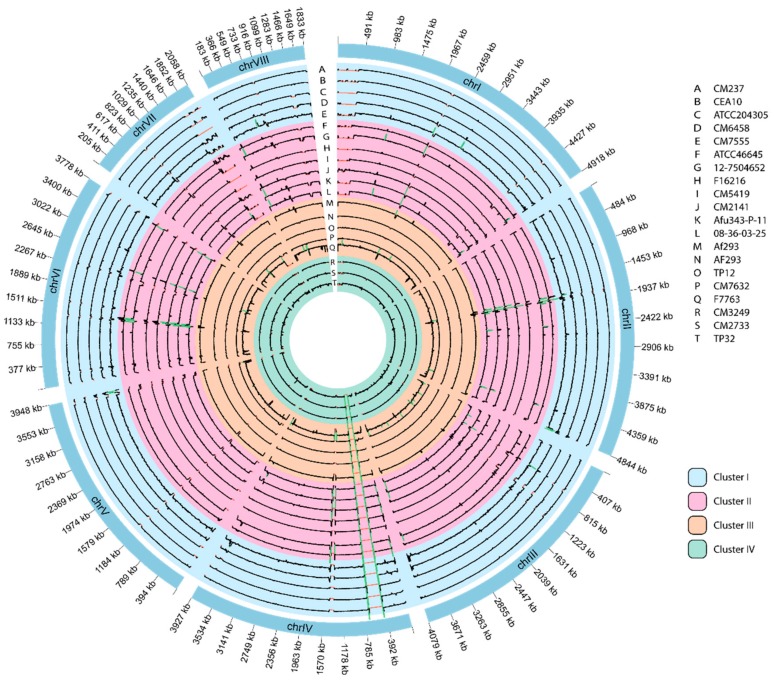
Circos representation of whole-genome depth of coverage of 20 *A. fumigatus* genomes, averaged over 10,000-bp bins. Red marks showed null coverage, black marks a range of coverage between 1–500, and green marks showed a coverage higher than 500. Color legend shows the selected genomes of each cluster: I (blue), II (pink), III (orange) and IV (green).

The depth of coverage from a selected group of whole-sequenced genomes was plotted in a Circos image to make an exploratory analysis, and to visualize similarities and differences in genome structure (Figure 3). None of the 20 *A. fumigatus* genomes included in this study displayed chromosomal or copy number variations. However, small deletions were observed in multiple chromosomes: regions smaller than 100 kbp seemed to be deleted in chromosomes 3, 4, 5, 6, 7 and 8. Also, what seemed to be large-scale deletions (bigger than 300 kbp) were detected in the edges of chromosomes 1 and 7 in genomes of cluster I and II. It is noteworthy that there was a huge red region in chromosome 4 present in all genomes marked as an expected deletion, even in the two different Af293 genomes downloaded and sequenced in-house although the reference genome used was the same Af293 strain genome.

### 3.7. Genome Comparisons Based on Their Mating Type

There were no differences in the strain genetic background between genomes harboring the mating type 1 or 2. In fact, strain genomes of both mating types were present in all clusters. In order to detect if there was any differential presence or absence of certain genes based on their particular mating type, all genomes were divided according to their mating type and mapped to both reference genomes (Af293, Mat1.2 and A1163, Mat1.1). No differences in the presence of genes were found in Mat1.1 strain genomes other than those encoded by the Mat1.2 locus itself (Mat1.2.1 and Mat1.2.4). The same situation was found within strains with Mat1.2 genomes; there were no differences in genes other than the gene Mat1.1.1 itself encoded by the Mat1.1 locus.

## 4. Discussion

Different typing techniques have demonstrated remarkable genomic diversity among strains of *A. fumigatus* [13,37]. *Aspergillus fumigatus* is found in multiple environmental niches which contribute to its genotypic and phenotypic heterogeneity among isolates [38]. Therefore, understanding the association between genomic diversity and different biological and phenotypic features, such as the spectrum of pathogenicity and antifungal resistance, among others, is a challenge for multiple *Aspergillus* laboratories. In this context, WGS is emerging as a promising tool for strain characterization [18,39]. Since WGS considers the entire genetic material of every isolate, it provides the highest discriminatory power that any technique can reach, allowing for the differentiation of closely related strains [39]. In addition, WGS could emerge as a useful tool for gene detection and other sequence-based investigations [9]. This source of an unprecedented amount of novel and comparative data could aid in the development of a huge database which would contain information about the biological differences between *A. fumigatus* strains, such as their origin, diversity and population structure. Previous WGS studies with a suitable depth of coverage had shown variation in genome structure with small-scale insertions and deletions, and recombination events among *A. fumigatus* isolates [40]. However, due to the complexity of biological systems, some difficulties need to be overcome in order to correlate complex biological traits with genomic features.

To date, most of the WGS studies are based on genome-scale comparisons in which the number of SNVs of each strain are obtained comparing its genome to a previously selected reference genome. In this work, we have made all comparison independently using two *A. fumigatus* reference genomes, Af293 and A1163. The differential grouping of the *A. fumigatus* population in four clusters is driven by the different SNVs found. In fact, the number of SNVs in each cluster and subcluster differed depending on the reference genome used to determine the number of variants. Particularly, the small number of SNVs found in cluster III when it was compared to Af293 reference genome is due to the fact that the genomes included in this cluster were more similar to Af293 and had twice the SNVs obtained with A1163 as a reference. Because cluster III was the smallest, formed by only five genomes, the WGS analysis would be improved by the addition of more genomes when available. In addition, it is important to highlight that the specific five SNVs in *cyp51A* present in all genomes belonging to cluster III (F46Y, M172V, N248T, D255E, and E427K) would have not been detected when Af293 genome was used as reference since those changes are also found in its genome. Other important changes could have been missed during the analysis depending on which genome was used as a reference, highlighting the importance of choosing an appropriate reference genome for each case.

The highest number of SNVs was found in cluster IV, which means that was the furthest and most different population compared to both references (Table 3). In fact, this result was in agreement with those computed using MEGA. Cluster IV was the most distant (in number of differences as well as in proportion) from all the subclusters (Tables S6 and S7). When the differences within subclusters were analyzed (Tables S8 and S9), this cluster had the greatest homogeneity. In conclusion, cluster IV is the most different *A. fumigatus* population compared to the others but also the most homogeneously composed. The results obtained by MEGA to study the evolutionary distances among genomes were in line with the results obtained from WGS phylogenetic studies, since the evolutionary distances supported the formed clusters and subclusters (Figure 1 and Figure 2, Tables S6 and S7).

Regarding the Circos visual representation of the depth of coverage (Figure 3), it is noteworthy that the described large-scale deletions were detected in the edges of chromosomes 1 and 7, so these possible deletion events could be confined to telomeric or subtelomeric regions. Notably, all cluster III genomes did not have these deletions, as well as the reference genome used, which belonged to this same cluster. Remarkably, a huge deletion in chromosome 4 was present in all genomes. This might be due to a misassembly or to sequencing errors since two different Af293 genomes (downloaded and sequenced in-house) were included in the analysis and also had this deletion in spite of being the same strain that the genome used as reference. Similar results have been found by other authors although no comments were made about it, probably because it was difficult to realize without sequencing in-house the Af293 [13]. Areas with higher depth of coverage might be a consequence of possible duplication events, pseudogene presence or artefacts. However, since these regions appeared specifically in chromosomes 2, 4 and 6 in most of the genomes of cluster II, it would be worthy to explore them further. A specific mapping approach should be developed to analyze all these regions and to determine if there are real duplications and also if they represent a hallmark for cluster characterization. Further research is in progress to clarify which genes are in these differential regions and which are their biological processes or functions in which they are involved.

Experimental investigations on *A. fumigatus* biology and virulence have historically used only a few particular strains, of which Af293 and A1163 are the most used. More than in any other comparisons, the genetic background must be highly considered in the genomes used as references, since it can be responsible for the variability in many biological aspects between isolates and thus responsible for different phenotypes. The first genome comparison study between Af293 and A1163 was accomplished in 2007, showing that both genomes were highly syntenic in most regions, having core genes highly conserved and with levels of identity of 99.8% at the genome level [18]. These results were confirmed later by Fedorova et al. [17]. Specifically, the Af293 genome contained 143 [17] to 208 unique genes [18], while the A1163, 218 [17] to 320 [18]. In both cases, more than 60% of the strain-specific genes were related to cellular metabolism, secondary metabolism, signaling and transcriptional regulation [17,18]. These sequence divergences could explain the phenotypic differences described by other authors who reported a high heterogeneity among these two isolates regarding abiotic stimuli physiological responses, levels of growth, and virulence [15,38,41], as well as in immunogenic responses [42]. The variability in the total amount of unique genes in each genome could be due to sequencing errors or, less probably, to new mutations generated during the repeated subculture [13]. These results are in agreement with our study, in which the Af293 and the A1163 reference genomes belonged to different phylogenetic clusters which means that there are considerable differences between both genomes. Therefore, the appropriate election of the most suitable reference genome in each context will ultimately result in a more appropriate comparison and lead to solid conclusions.

Apart from the differences already discussed between Af293 and A1163, comparative analyses of the mating loci showed that both strains belonged to opposite mating types [40,43]. Mating type genes are not true idiomorphs, as they occupy adjacent positions on the chromosome. Mat1.1 strains have only one gene in this locus that encodes a transcriptional factor with an alpha box domain (AFUB_042900, Mat1.1.), while Mat1.2 strains contain a truncated copy of the high mobility group (HMG) box (Afu3g06170, Mat1.2.1) and another gene (Afu3g06160, Mat1.2.4) needed for heterokaryon formation as part of the mating process [44,45,46]. In this study we also checked if there were gene differences (presence or absence) other than those responsible for the mating type (Mat1.1, Mat1.2.1 and Mat1.2.4) among both groups of *A. fumigatus* genomes using an innovative approach (Figure S1). No gene absences were found in Mat1.1 strain genomes other than those encoded by the Mat1.2 locus itself (Mat1.2.1 and Mat1.2.4). Some previous works have reported differential gene presence between strains that coincidentally had different mating types [17,18]. However, the differences found must be due to their own different genetic background and not because of their opposite mating type.

One of the main concerns of invasive aspergillosis treatment is the global emergence of azole resistance. Regarding azole resistance mechanisms, Cyp51A protein is the target of these drugs and the *cyp51A* gene is a hotspot for mutations that confer azole resistance. The most common *cyp51A* modifications could be grouped into two categories: *A. fumigatus* strains that harbor *cyp51A* single point mutations (G54, G138, M220, or G448) [47,48,49,50], and isolates with specific point mutations in *cyp51A* gene together with various size tandem repeat (TR) integrations in the promoter of the gene (TR34/L98H, TR34/L98H/S297T/F495I, TR46/Y121F/T289A, or TR53) [24,51,52,53]. Moreover, all these *cyp51A* modifications have been described to evolve from two different azole resistance acquisition routes: the first set of strains come from the clinical setting as a consequence of the in-host drug adaptation after azole exposure in the patient [54], while the second set of isolates with TR insertions are hypothesized to develop from the azole exposure in the environmental setting [13,37,55,56,57,58]. Thanks to the high amount of isolates harboring different azole resistance mechanisms included in the study, a clear tendency in terms of grouping can be observed. In agreement with previous studies [13,37,55,56,58,59,60], all genomes included in our work that harbor a TR integration with single point mutations in *cyp51A* gene were grouped in cluster II, while isolates with punctual modifications in this gene were spread across clusters I and II. The same happened with *cyp51A*-WT strains that were distributed across those clusters. Therefore, our study supported the idea that isolates with punctual modifications in *cyp51A* (G54, G138, M220, or G448) and also *cyp51A*-WT strains have a greater genetic diversity than TR azole resistant isolates (TR34/L98H, TR34/L98H/S297T/F495I), which reinforces the previously suggested idea that TR resistance mechanisms have developed from a reduced set of clonally related strains with shorter genetic distances among them [59,60]. In this study, only genomes from strains which harbor the TR34/L98H resistance mechanism (with or without the S297T and F495I modifications) have been included. It would be most interesting to include isolates harboring TR46/Y121F/T289A, and TR53 azole resistance mechanisms. Furthermore, it is noteworthy that cluster III and IV were formed specifically by particular isolates that harbor 3 or 5 *cyp51A* modifications (F46Y, M172V, E427K and F46Y, M172V, N248T, D255E, E427K, respectively). This result was in agreement with previous studies developed in our group [19]. Further characterization of clado-specific genes would enhance our understanding of antifungal resistance mechanisms and help to unravel the environmental route resistance development.

Despite all these known azole resistance mechanisms, there is an increasing number of *A. fumigatus* azole resistant isolates for which the underlying mechanism remains unknown [61,62] or which are independent of *cyp51A* modifications [63]. The availability of *A. fumigatus* genomes to compare closely related strains but with differences in their azole susceptibility phenotype will increase the chance of finding genetic differences that could explain the phenotypic variations.

In conclusion, a great amount of novel and useful information can be derived from WGS studies. Here we particularly demonstrated that the selection of strains and reference genomes is crucial for comparative analysis at genomic and phenotypic level. Further compilation of *A. fumigatus* data generated by WGS studies could greatly enhance the understanding of molecular mechanisms involved in antifungal drug resistance and its development, as well as in many other biological functions that remain unknown. As the number of *A. fumigatus* genomes have increasingly been publically available, researchers will be able to increase the possibilities for data analysis which will ultimately allow them to come up with possible conclusions about an infinite number of hypotheses.

## Figures and Tables

**Table 1:** *Aspergillus fumigatus* strains whole genome sequenced in our laboratory. The assigned lineage was based on the phylogenetic study.

Samples	Origin	*cyp51A* Modifications	AZL SC	Mating Type	Source	Lineage
AF293	SP	5SNPs	S	M1.2	CL	3
*akuB* ^KU80^	SP	WT	S	M1.1	CL	1.1
ATCC204305	SP	I242V	S	M1.1	CL	1.2
ATCC46645	SP	WT	S	M1.1	CL	2.1
CEA10	SP	WT	S	M1.1	CL	1.1
CM2141	SP	WT	S	M1.1	CL	2.2
CM237	SP	WT	S	M1.1	CL	1.1
CM2495	SP	3SNPs	S	M1.1	CL	4
CM2730	SP	3SNPs	S	M1.2	CL	4
CM2733	SP	3SNPs	S	M1.1	CL	4
CM3248	SP	N248K	S	M1.1	CL	1.1
CM3249	SP	3SNPs	S	M1.1	CL	4
CM3249b	SP	3SNPs	S	M1.1	CL	4
CM3262	SP	3SNPs	S	M1.1	CL	4
CM3720	SP	3SNPs	S	M1.1	CL	4
CM4602	SP	3SNPs	S	M1.2	CL	4
CM4946	SP	3SNPs	S	M1.2	CL	4
CM5419	SP	WT	S	M1.2	CL	2.2
CM5757	SP	WT	S	M1.2	CL	1.1
CM6126	SP	WT	S	M1.2	CL	1.1
CM6458	SP	WT	S	M1.2	CL	1.3
CM7510	SP	WT	R	M1.1	CL	1.1
CM7555	SP	WT	R	M1.2	CL	1.3
CM7560	SP	3SNPs	S	M1.1	CL	4
CM7570	SP	3SNPs	S	M1.1	CL	4
CM7632	SP	5SNPs	S	M1.1	CL	3
TP12	SP	5SNPs	S	M1.2	CL	3
TP32	SP	3SNPs	S	M1.1	CL	4

SP (Spain), SNPS (single nucleotide polymorphisms), 3SNPs *cyp51A* modifications (F46Y, M172V, E427K) and 5SNPs *cyp51A* modifications (F46Y, M172V, N248T, D255E, E427K), AZL susceptibility (azole susceptibility), S (susceptible), R (resistant), CL (clinical).

**Table 2:** *Aspergillus fumigatus* genomes from public data bases included in this study. The assigned lineage is based on the phylogenetic study.

Samples	Origin	*cyp51A* Modifications	AZL SC	Mating Type	Source	Lineage	Ref.
08-12-12-13	NT	TR_34_/L98H, S297T, F495I	R	M1.1	CL	2.2	[13]
08-19-02-10	NT	TR_34_/L98H	R	M1.2	ENV	2.2	[13]
08-19-02-30	NT	WT	S	M1.1	ENV	1.3	[13]
08-19-02-46	NT	TR_34_/L98H	R	M1.1	ENV	2.2	[13]
08-19-02-61	NT	TR_34_/L98H	R	M1.1	ENV	2.2	[13]
08-31-08-91	NT	TR_34_/L98H	R	M1.1	CL	2.2	[13]
08-36-03-25	NT	TR_34_/L98H, S297T, F495I	R	Unclear	CL	2.2	[13]
09-7500806	UK	WT	S	M1.2	CL	1.3	[13]
10-01-02-27	NT	TR_34_/L98H	R	M1.2	CL	2.2	[13]
12-7504462	UK	WT	S	M1.2	CL	1.3	[13]
12-7504652	UK	WT	S	M1.1	CL	2.1	[13]
12-7505054	UK	WT	S	M1.1	CL	2.1	[13]
12-7505220	UK	TR_34_/L98H	R	M1.1	CL	2.2	[13]
12-7505446	UK	TR_34_/L98H	R	M1.2	CL	2.2	[13]
Af293	UK	5SNPs	S	M1.2	CL	3	[13]
AF41	UK	WT	S	M1.2	CL	1.1	[13]
Af65	UK	WT	S	M1.2	CL	2.1	[13]
AF72	UK	G54E	R	M1.2	CL	1.1	[13]
AF90	UK	M220V	R	M1.1	CL	1.1	[13]
Afu1042-09	IN	TR_34_/L98H	R	M1.1	CL	2.2	[13]
Afu124-E11	IN	TR_34_/L98H	R	M1.1	ENV	2.2	[13]
Afu166-E11	IN	TR_34_/L98H	R	M1.1	ENV	2.2	[13]
Afu218-E11	IN	TR_34_/L98H	R	M1.1	ENV	2.2	[13]
Afu257-E11	IN	TR_34_/L98H	R	M1.1	ENV	2.2	[13]
Afu343-P-11	IN	TR_34_/L98H	R	Unclear	CL	2.2	[13]
Afu591-12	IN	TR_34_/L98H	R	M1.1	CL	2.2	[13]
Afu942-09	IN	TR_34_/L98H	R	M1.1	CL	2.2	[13]
F12041	UK	G138C	R	M1.2	CL	2.1	[21] *
F12219	UK	G54R	R	M1.2	CL	1.1	[21] *
F12636	UK	G54E	R	M1.1	CL	2.1	[21] *
F13535	UK	G138C	R	M1.2	CL	2.1	[21] *
F13619	UK	H147Y, G448S	R	M1.1	CL	2.1	[21] *
F13952	UK	G138C	R	M1.2	CL	2.1	[21] *
F14403	UK	G54R	R	M1.1	CL	1.1	[21] *
F14513G	UK	G138C	R	M1.2	CL	2.1	[21] *
F14532	UK	M220T	R	M1.1	CL	2.1	[21] *
F14946G	UK	WT	S	M1.2	CL	1.1	[21] *
F15390	UK	M220T	R	M1.1	CL	2.1	[21] *
F15927	CN	3SNPs	S	M1.1	CL	4	[21] *
F16134	DN	M220K	R	M1.2	CL	1.1	[21] *
F16216	UK	TR_34_/L98H	R	M1.2	CL	2.1	[21] *
F16311	UK	WT	S	M1.1	CL	1.1	[21] *
F17582	UK	WT	S	M1.2	CL	2.1	[21] *
F17729	UK	WT	S	M1.2	CL	1.1	[21] *
F17729W	UK	WT	S	M1.2	CL	1.1	[21] *
F17764	CN	WT	S	M1.1	CL	1.1	[21] *
F18085	UK	WT	S	M1.1	CL	1.1	[21] *
F5211G	UK	WT	S	M1.1	CL	2.1	[21] *
F7763	UK	5SNPs	S	M1.1	CL	3	[21] *
IFM55369	JP	WT	S	M1.1	CL	1.2	[22]
IFM58026	JP	N248K	S	M1.2	CL	1.1	[22]
IFM58029	JP	WT	S	M1.2	CL	1.2	[22]
IFM58401	JP	WT	S	M1.2	CL	1.1	[22]
IFM59056	JP	WT	S	M1.1	CL	1.1	[22]
IFM59073	JP	WT	S	M1.2	CL	1.2	[22]
IFM59359	JP	WT	S	Unclear	CL	1.2	[22]
IFM59361	JP	WT	S	M1.1	CL	1.3	[22]
IFM59365	JP	WT	S	M1.2	CL	1.1	[22]
IFM59777	JP	WT	S	M1.1	CL	1.2	[22]
IFM60514	JP	WT	S	M1.2	CL	1.1	[22]
IFM61118	JP	N248K	S	M1.1	CL	1.1	[22]
IFM61407	JP	WT	S	Unclear	CL	1.1	[22]
IFM61578	JP	P216L	S	M1.2	CL	1.1	[22]
IFM61610	JP	WT	S	M1.2	CL	1.1	[22]
IFM62516	JP	P329P	S	M1.1	CL	1.2	[22]
SL143435	PT	N248K	S	M1.2	UNK	1.1	*
SL143436	PT	WT	S	M1.2	UNK	1.1	*
SL143437	PT	WT	S	M1.1	UNK	1.3	*
SL143438	PT	N248K	S	M1.2	UNK	1.1	*
SL143439	PT	WT	S	M1.1	UNK	1.3	*
SL143440	PT	3SNPs	S	M1.1	UNK	4	*
SL143441	PT	WT	S	M1.1	UNK	1.1	*
SL146112	PT	N248K	S	M1.2	UNK	1.1	*

NT (the Netherlands), UK (the United Kingdom), IN (India), CN (Canada), JP (Japan), PT (Portugal). SNPs (single nucleotide polymorphisms), 3SNPs *cyp51A* modifications (F46Y, M172V, E427K) and 5SNPs *cyp51A* modifications (F46Y, M172V, N248T, D255E, E427K), AZL SC (azole susceptibility), S (susceptible), R (resistant), CL (clinical), ENV (environmental), UNK (unknown), * NCBI SRA.

**Table 3:** Mean of single nucleotide variants (SNVs). Variants within clusters and subclusters compared to both reference genomes.

Phylogenetic Groups	SNVs vs. A1163 Reference Genome	SNVs vs. Af293 Reference Genome
Subcluster I.1	34,544	75,653
Subcluster I.2	40,124	65,121
Subcluster I.3	46,811	79,899
Subcluster II.1	81,478	98,423
Subcluster II.2	80,994	97,749
Cluster III	82,235	35,268
Cluster IV	158,154	159,742

**Table 4:** Single nucleotide variant differences compared to both reference genomes. Variants of each *A. fumigatus* genome included in this study.

Samples	A1163	Af293	Samples	A1163	Af293	Samples	A1163	Af293
08-12-12-13	80,791	91,956	CM2495	151,089	148,524	F17582	79,598	97,024
08-19-02-10	75,401	89,762	CM2730	157,505	159,177	F17729	44,054	79,106
08-19-02-30	42,487	87,831	CM2733	154,361	160,494	F17729W	43,663	78,416
08-19-02-46	78,449	96,243	CM3248	22,834	74,568	F17764	25,812	70,013
08-19-02-61	80,244	96,416	CM3249	157,242	159,900	F18085	51,241	78,553
08-31-08-91	88,972	98,911	CM3249b	154,510	157,780	F5211G	82,091	99,726
08-36-03-25	83,921	102,006	CM3262	161,695	160,926	F7763	84,764	55,332
09-7500806	45,286	74,134	CM3720	156,600	158,781	IFM55369	42,849	64,003
10-01-02-27	72,487	93,299	CM4602	155,381	161,767	IFM58026	22,186	68,777
12-7504462	48,927	75,301	CM4946	158,692	161,082	IFM58029	36,521	63,106
12-7504652	80,203	93,323	CM5419	89,354	99,771	IFM58401	26,669	63,489
12-7505054	86,952	101,471	CM5757	36,027	74,420	IFM59056	30,841	61,481
12-7505220	75,479	100,229	CM6126	55,871	86,902	IFM59073	43,230	68,775
12-7505446	86,194	100,736	CM6458	42,482	71,670	IFM59359	39,309	67,118
Af293	83,486	313	CM7510	33,559	79,283	IFM59361	42,795	73,228
AF293	78,045	659	CM7555	51,657	77,679	IFM59365	21,830	74,248
AF41	34,879	72,914	CM7560	158,657	153,769	IFM59777	40,117	65,300
Af65	90,143	103,614	CM7570	160,908	162,292	IFM60514	39,629	75,765
AF72	35,271	73,632	CM7632	81,519	25,490	IFM61118	21,905	70,230
AF90	40,262	73,395	F12041	78,812	93,712	IFM61407	32,025	76,034
Afu1042-09	80,346	96,348	F12219	35,440	73,872	IFM61578	26,201	67,946
Afu124-E11	81,115	97,509	F12636	80,359	98,501	IFM61610	41,690	74,102
Afu166-E11	80,948	97,350	F13535	78,741	93,777	IFM62516	41,229	67,364
Afu218-E11	80,571	96,955	F13619	85,412	107,985	SL143435	22,037	81,031
Afu257-E11	80,499	97,008	F13952	78,466	93,926	SL143436	52,604	84,188
Afu343-P11	73,797	101,305	F14403	27,476	74,454	SL143437	61,289	91,394
Afu591-12	80,781	97,039	F14513G	78,647	94,213	SL143438	22,676	81,649
Afu942-09	80,827	97,149	F14532	79,931	102,436	SL143439	39,562	87,955
*akuB* ^KU80^	1043	78,152	F14946G	44,442	79,719	SL143440	162,993	165,788
ATCC 204305	37,614	60,180	F15390	79,743	101,967	SL143441	38,498	72,994
ATCC 46645	84,994	103,941	F15927	161,157	164,076	SL146112	21,782	81,137
CEA10	1009	75,854	F16134	19,660	77,718	TP12	83,363	24,982
CM2141	88,704	107,238	F16216	78,073	90,736	TP32	163,367	162,032
CM237	48,402	83,874	F16311	51,387	78,630	F17582	79,598	97,024

**Table 5:** Types of SNVs and features. Variants found within each cluster compared to both genomes.

Variants	A1163 Reference Genome				Af293 Reference Genome			
	I	II	III	IV	I	II	III	IV
Frameshift variants	256	512	477	828	446	587	133	819
Inframe deletions	149	278	264	511	246	330	94	527
Inframe insertions	191	369	291	561	325	447	91	556
Intergenic variants	6405	11,107	12,819	6730	13,428	16,394	3,785	8792
Intron variants	1568	3668	3418	9066	3120	4322	960	8670
Missense variants	4342	10,251	10,008	23,107	8766	11,973	2562	22,746
Protein altering	8	19	12	18	16	22	4	21
Start lost	9	16	23	57	21	24	6	57
Stop gained	95	230	264	298	141	218	43	288
Stop lost	13	27	32	61	25	33	9	61
Stop retained	10	22	21	52	17	24	5	52
Synonymous variants	3675	9026	9005	26,080	8189	11,058	2365	25,418

## Supplementary Materials

The following are available online at http://www.mdpi.com/2073-4425/9/7/363/s1, Figure S1: Workflow for obtaining genes present in all Mat1.1 samples but not present in Mat1.2 (A); and on the contrary (B). Table S1: Mapping rate and coverage at 10× of 101 *A. fumigatus* strains against both reference genomes. Table S2: MEGA genetic distance estimations: pairwise distance matrix calculating the proportion of differences (p-model) and using Af293 as reference genome. Table S3: MEGA genetic distance estimations: pairwise distance matrix calculating the number of differences and using Af293 as reference genome. Table S4: MEGA genetic distance estimations: pairwise distance matrix calculating the proportion of differences (p-model) and using A1163 as reference genome. Table S5: MEGA genetic distance estimations: pairwise distance matrix calculating the number of differences and using A1163 as reference genome. Table S6: MEGA genetic distance estimations: mean distances within subclusters calculating the proportion and number of differences and using Af293 as reference genome. Table S7: MEGA genetic distance estimations: mean distances within subclusters calculating the proportion and number of differences and using A1163 as reference genome. Table S8: MEGA genetic distance estimations: matrix of the mean distances between subclusters calculating the proportion and number of differences and using Af293 as reference genome. Table S9: MEGA genetic distance estimations: matrix of the mean distances between subclusters calculating the proportion and number of differences and using A1163 as reference genome.
