# Supplementary figures and images for: Genome-Wide Comparative Analysis of Aspergillus fumigatus Strains: The Reference Genome as a Matter of Concern
Source: Genes (Basel). 2018 Jul 19;9(7):363. doi: 10.3390/genes9070363 (PMC6071029; doi:10.3390/genes9070363)

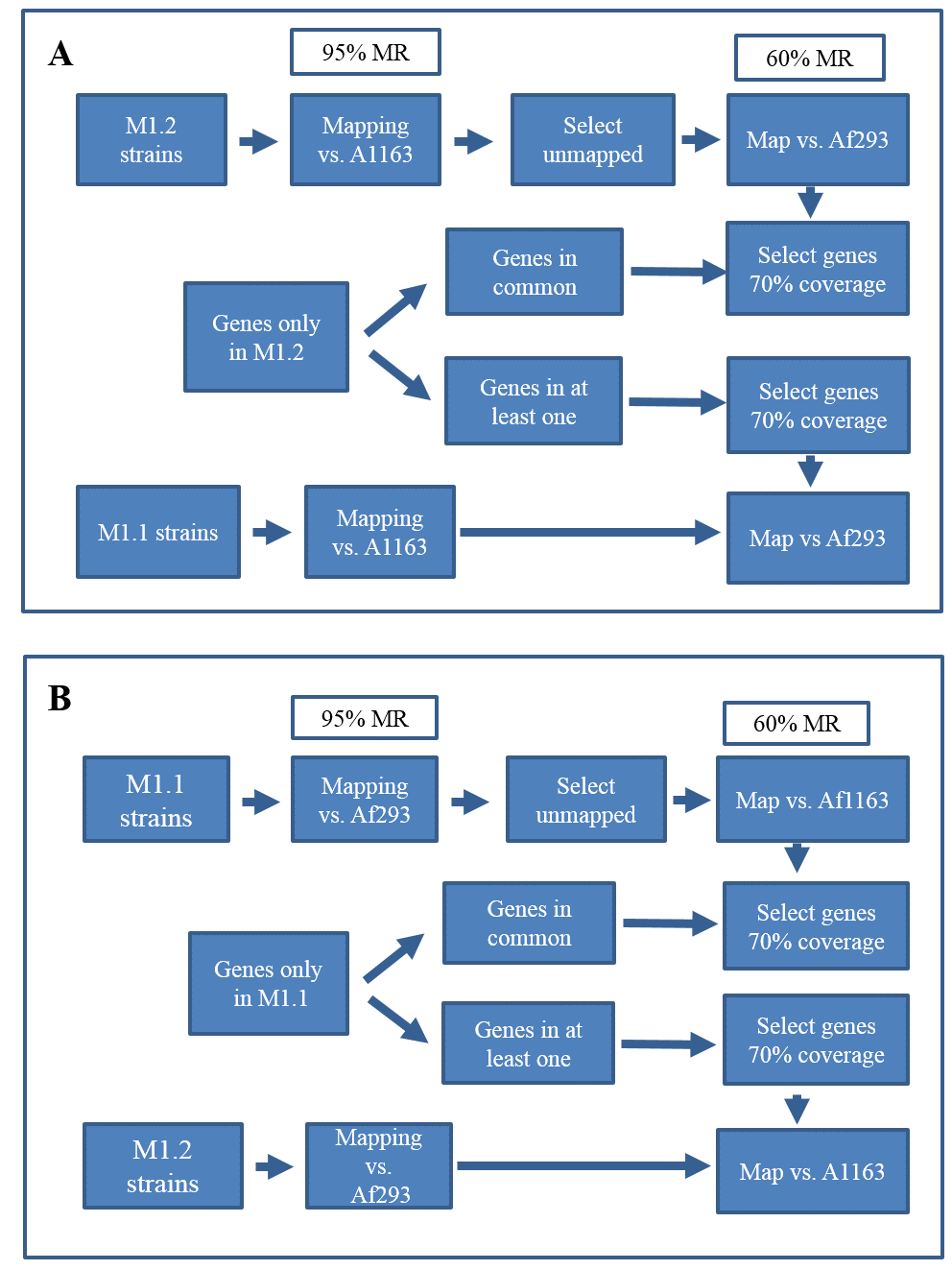

Supplement: Supplementary file 1 [file genes-09-00363-s001.zip › Figure S1.tiff]
